# Supplementary figures and images for: Microstructural and functional gradients are increasingly dissociated in transmodal cortices
Source: PLoS Biol. 2019 May 20;17(5):e3000284. doi: 10.1371/journal.pbio.3000284 (PMC6544318; doi:10.1371/journal.pbio.3000284)

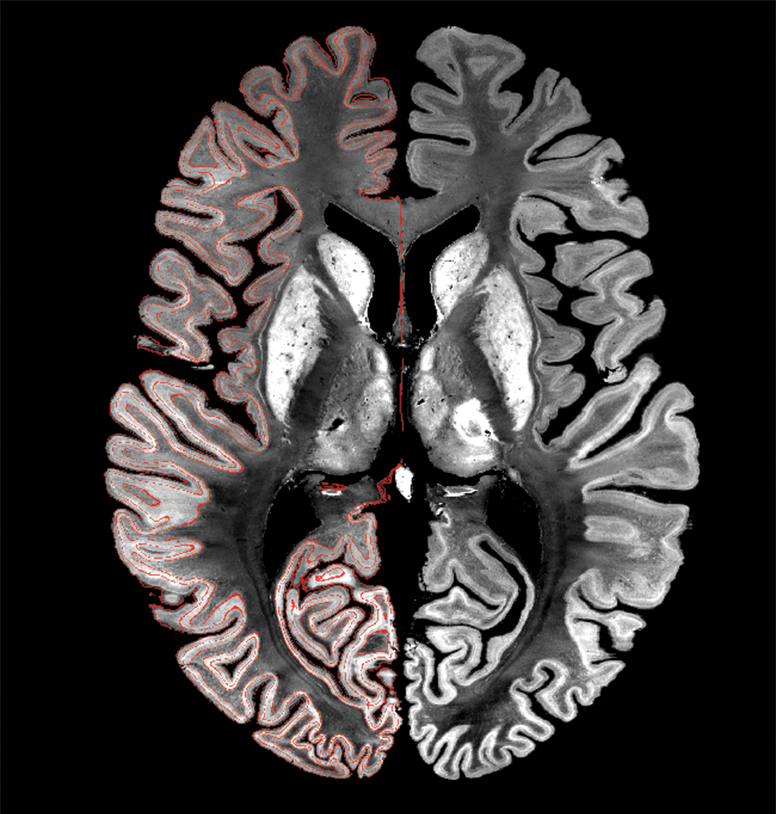

Supplement: S1 Fig — Notably, we corrected for the linear relationship between intensity values and midsurface y coordinate (r = −0.68, P < 0.001), which existed due to coronal slicing and reconstruction of the BigBrain. Histological data is openly available as part of the BigBrain initiative (https://bigbrain.loris.ca/main.php). (TIF) [file pbio.3000284.s001.tif]

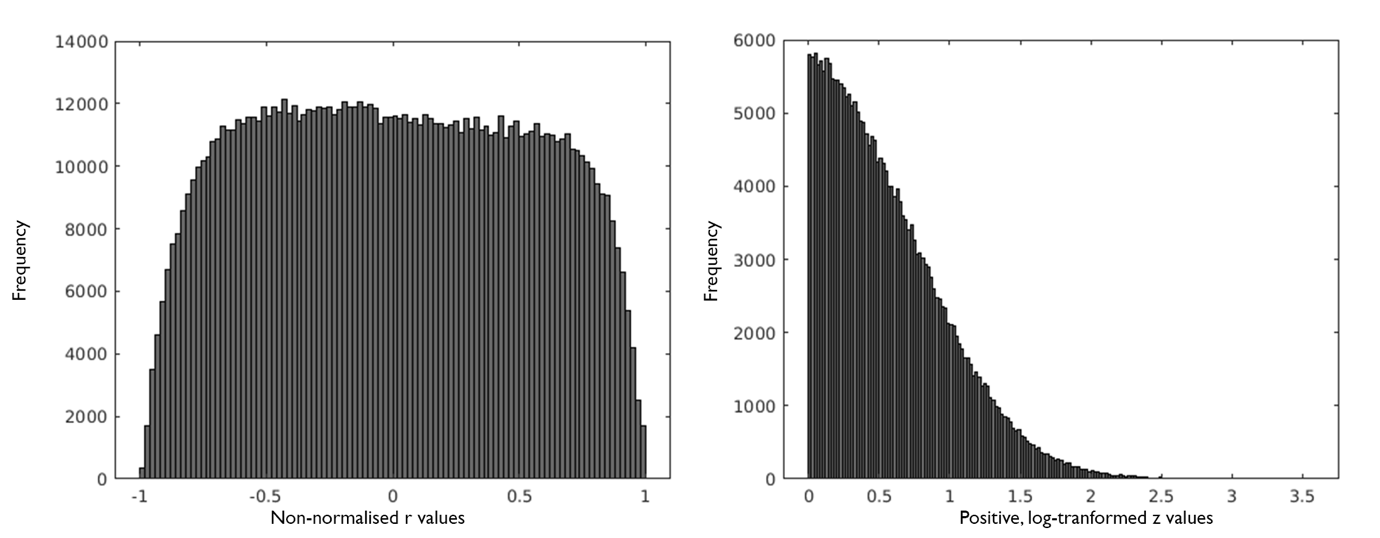

Supplement: S2 Fig — (Left) Frequency of r values calculated by Pearson product moment correlation coefficient of the nodal intensity profiles, controlling for the average intensity profile. (Right) Frequency of positive z values following log transformation of r values. Histological data is openly available as part of the BigBrain initiative (https://bigbrain.loris.ca/main.php). HIST, histology-based; MPC, microstructure profile covariance. (TIF) [file pbio.3000284.s002.tif]

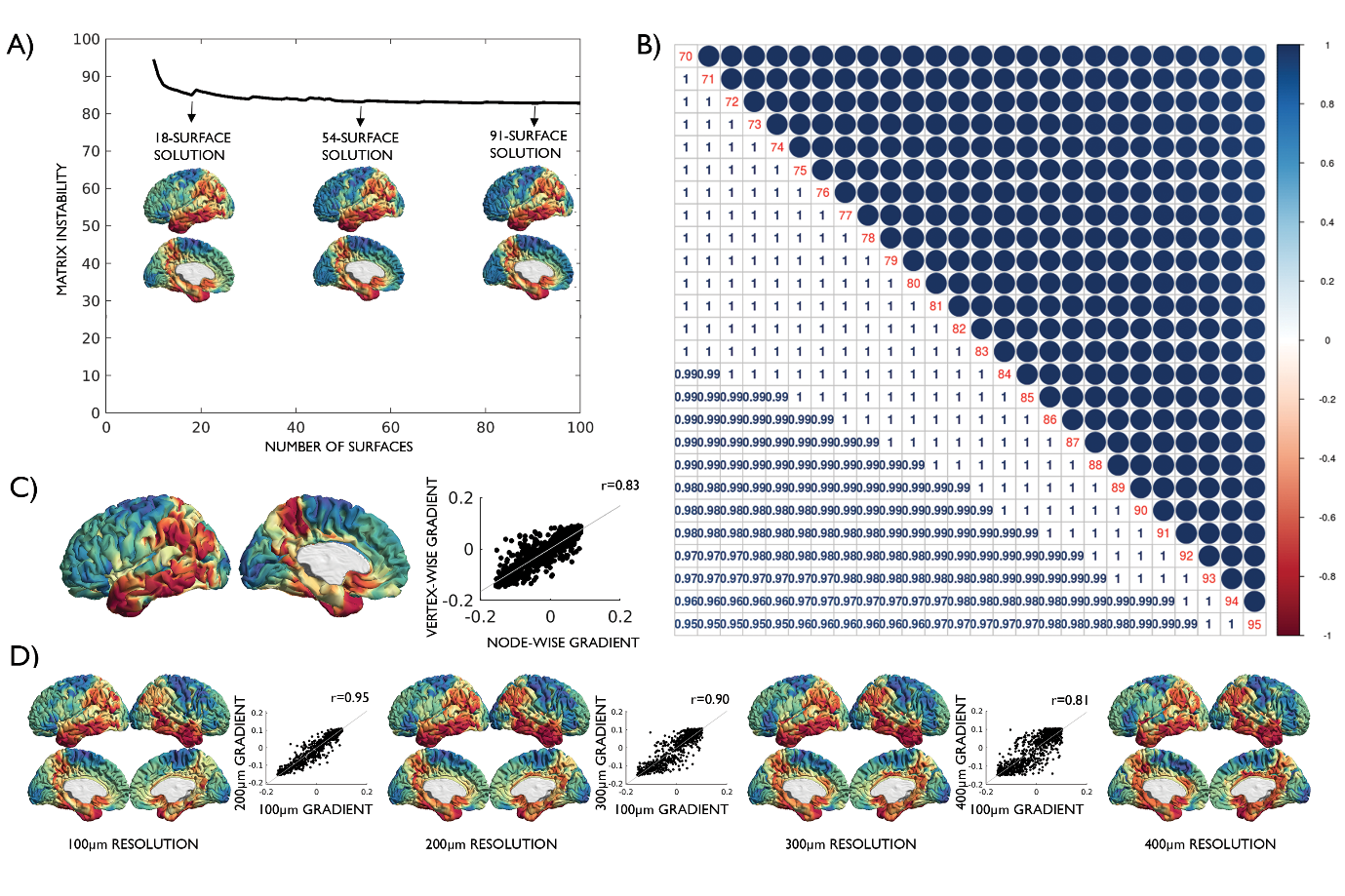

Supplement: S3 Fig — (A) MPCHIST matrix instability using between 10 and 100 intracortical surfaces. G1HIST was consistent regardless of the number of intracortical surfaces used, as shown by the strong spatial correlation of the 18-, 54- and 91-surface solutions (all r > 0.97, all P < 0.001). (B) Correlation matrix depicting the high correspondence of G1HIST solutions with 70–95% row-wise matrix thresholding (0.95 < r < 1, all P < 0.001). (C) Estimation of G1HIST from 20488 vertices resulted in a consistent G1HIST to the 1,012 parcel construction pipeline (r = 0.87, P < 0.001). (D) The 200-μm, 300-μm, and 400-μm resolution BigBrain data sets were characterised as lower resolution replications, and G1HIST was found to be highly correlated across these resolutions (all r > 0.81, all P < 0.001). Histological data is openly available as part of the BigBrain repository (https://bigbrain.loris.ca/main.php). G1, first principal gradient; HIST, histology-based; MPC, microstructure profile covariance. (TIF) [file pbio.3000284.s003.tif]

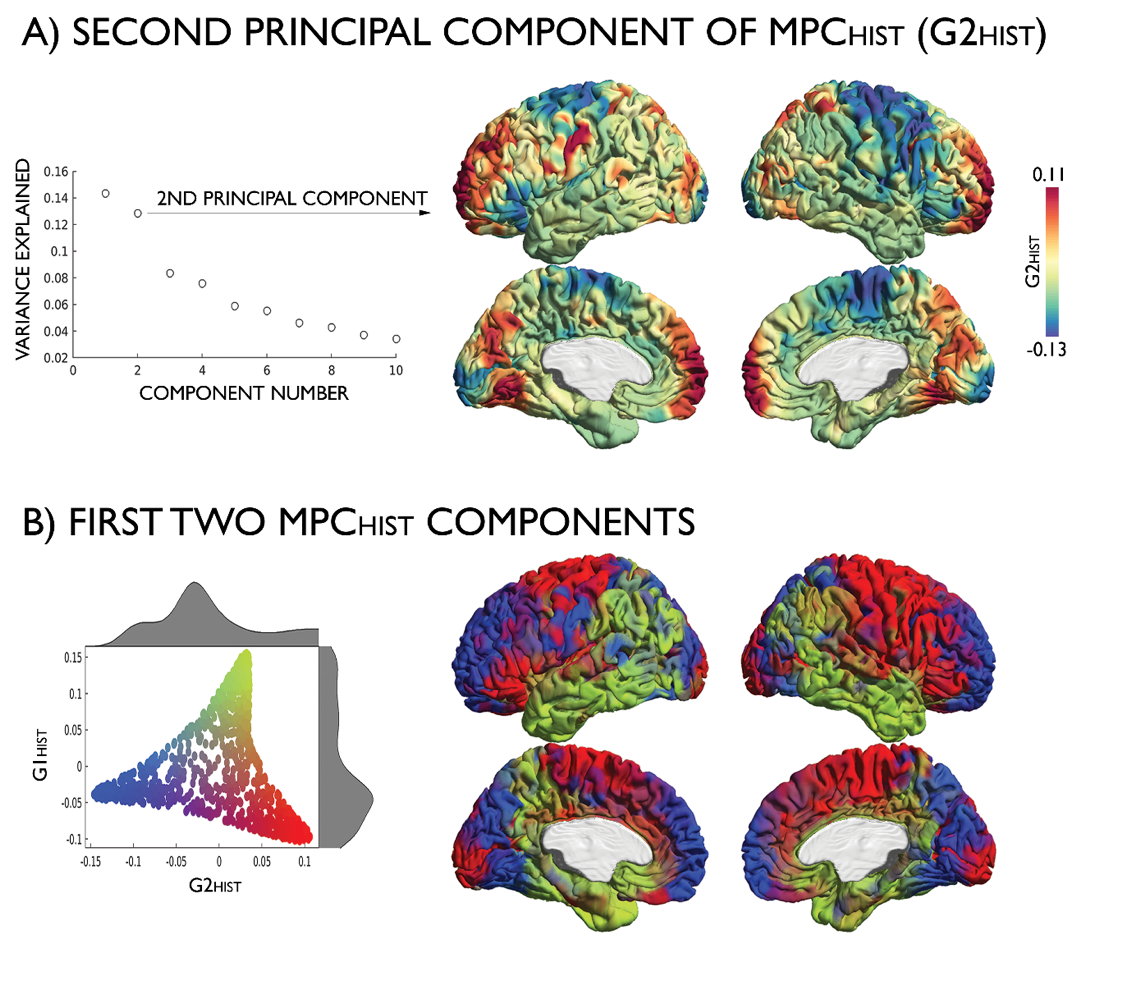

Supplement: S4 Fig — (A) The second principal component, accounting for 12.7% of variance in MPCHIST components, is projected on the BigBrain midsurface. (B) Scatterplot depicting the first two embedding gradients, with corresponding probability density functions. The second gradient divides the lower-order areas of the first gradient, insomuch that somatomotor and primary visual areas (red) are separated from ventral prefrontal areas and secondary visual areas (blue). Histological data is openly available as part of the BigBrain repository (https://bigbrain.loris.ca/main.php). HIST, histology-based; MPC, microstructure profile covariance. (TIF) [file pbio.3000284.s004.tif]

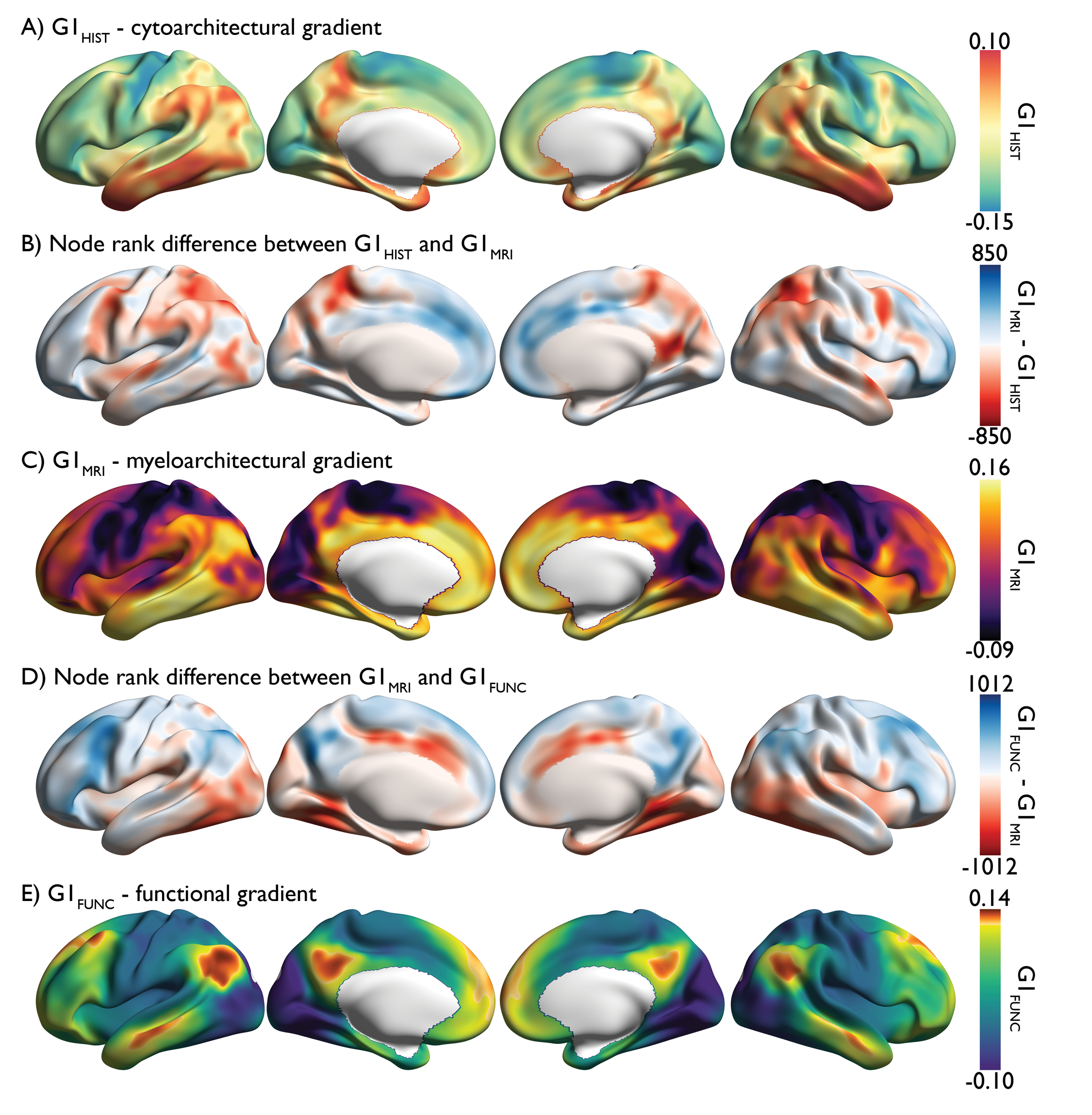

Supplement: S5 Fig — Histological data is openly available as part of the BigBrain repository (https://bigbrain.loris.ca/main.php). In vivo imaging data is openly available as part of the HCP S900 release (https://www.humanconnectome.org/study/hcp-young-adult/document/900-subjects-data-release). HCP, Human Connectome Project. (TIF) [file pbio.3000284.s005.tif]

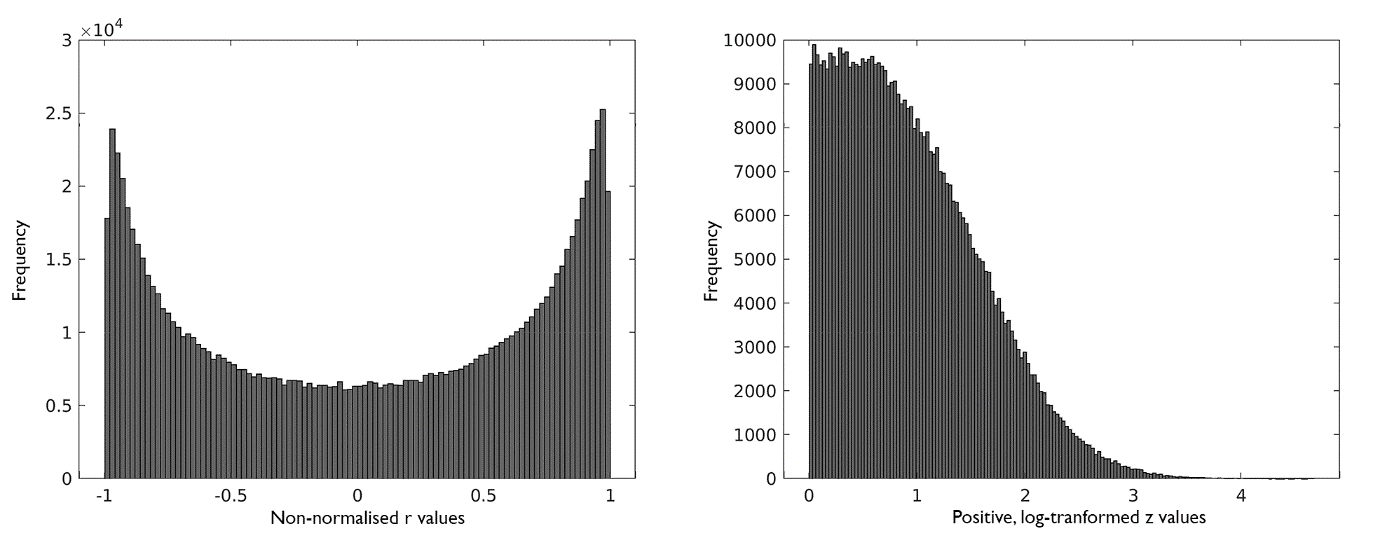

Supplement: S6 Fig — (Left) Frequency of r values calculated by Pearson product–moment correlation coefficient of the nodal intensity profiles, controlling for the average intensity profile. (Right) Frequency of positive z values following log transformation of r values. In vivo imaging data is openly available as part of the HCP S900 release (https://www.humanconnectome.org/study/hcp-young-adult/document/900-subjects-data-release). HCP, Human Connectome Project; MPC, microstructure profile covariance; MRI, magnetic resonance imaging. (TIF) [file pbio.3000284.s006.tif]

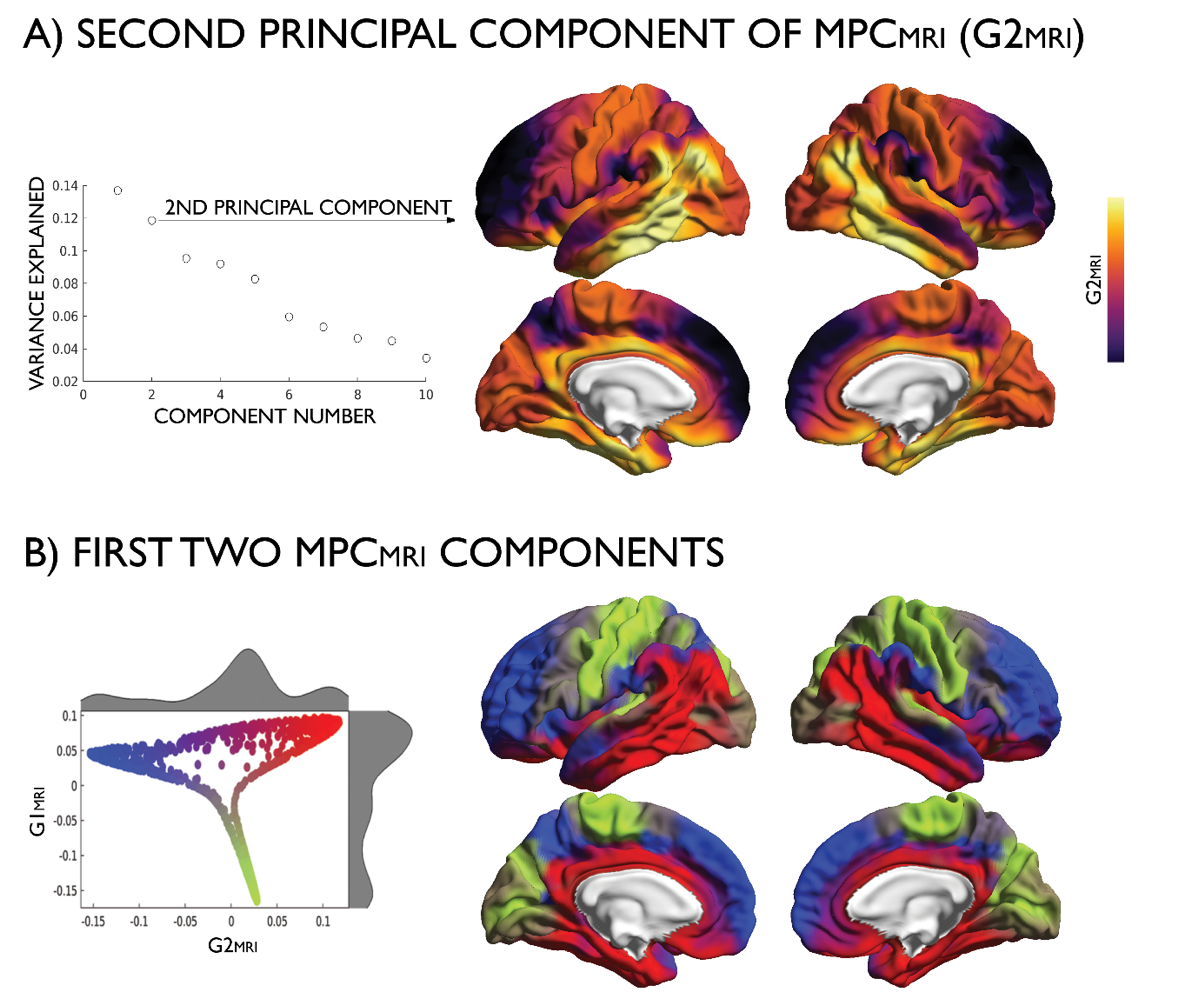

Supplement: S7 Fig — (A) The second principal component, accounting for 11.7% of variance in MPCMRI components, projected on the conte69 midsurface. (B) Scatterplot depicting the first two embedding gradients, with corresponding probability density functions. The second gradient divides the higher-order areas of the first gradient, insomuch that the cingulate, orbitofrontal cortex and the inferior temporal gyrus (red) are separated from the prefrontal cortex, precuneus, temporo–parietal junction, and superior temporal gyrus (blue). In vivo imaging data is openly available as part of the HCP S900 release (https://www.humanconnectome.org/study/hcp-young-adult/document/900-subjects-data-release). HCP, Human Connectome Project; MPC, microstructure profile covariance; MRI, magnetic resonance imaging. (TIF) [file pbio.3000284.s007.tif]

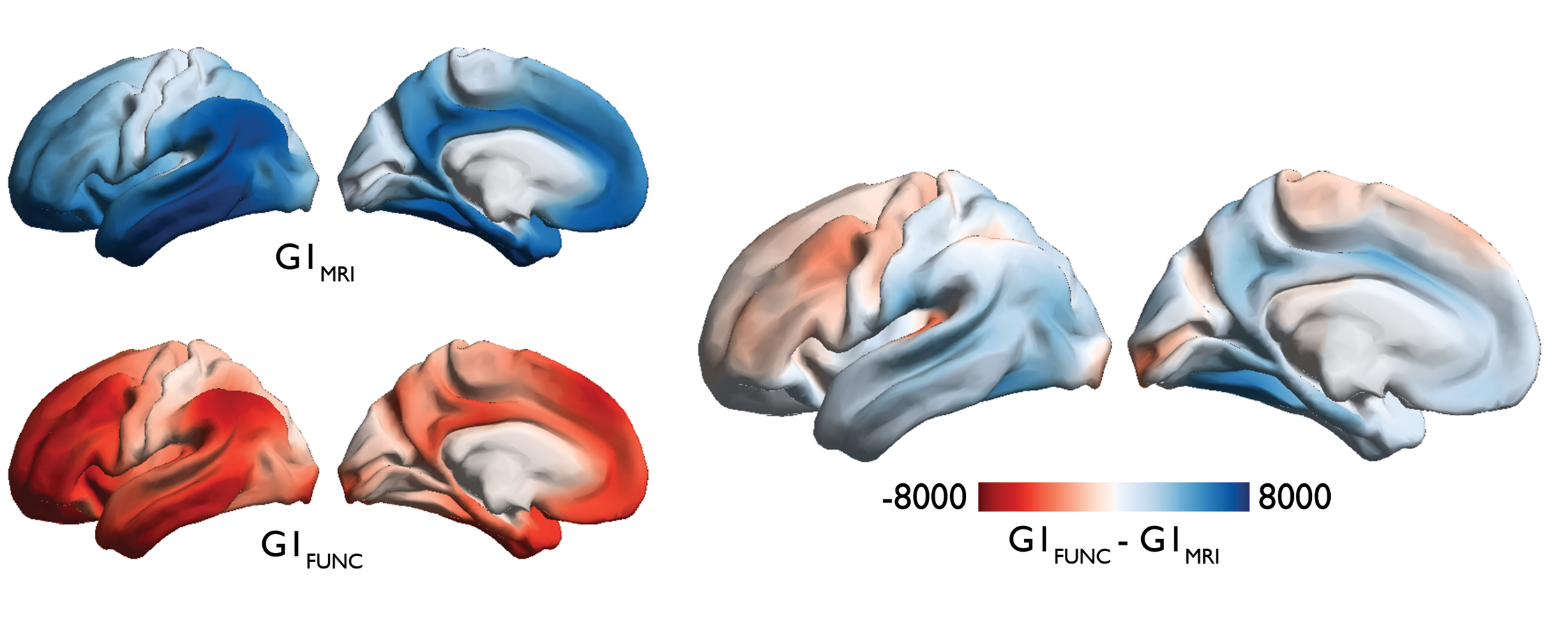

Supplement: S8 Fig — In vivo imaging data is openly available as part of the HCP S900 release (https://www.humanconnectome.org/study/hcp-young-adult/document/900-subjects-data-release). FUNC, functional; G1, first principal gradient; HCP, Human Connectome Project; MRI, magnetic resonance imaging. (TIF) [file pbio.3000284.s008.tif]

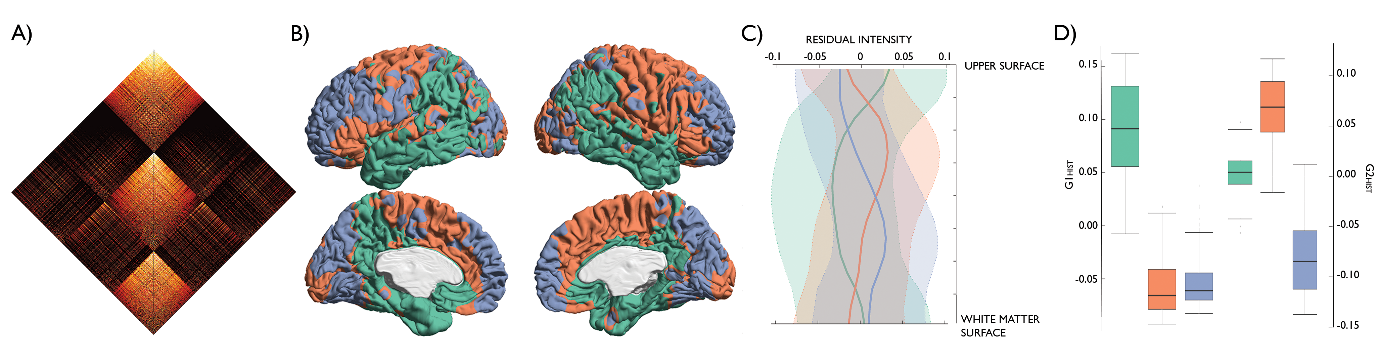

Supplement: S9 Fig — (A) MPCHIST matrix sorted by community membership. (B) Modular decomposition of MPCHIST projected on the BigBrain midsurface. (C) Mean ± SD of residual intensity profile for each module, after correction for the midsurface y coordinate and demeaning. (D) Boxplot depicts the unique positions of modules along the first two principal gradients. Histological data is openly available as part of the BigBrain repository (https://bigbrain.loris.ca/main.php). HIST, histology-based; MPC, microstructure profile covariance. (TIF) [file pbio.3000284.s009.tif]

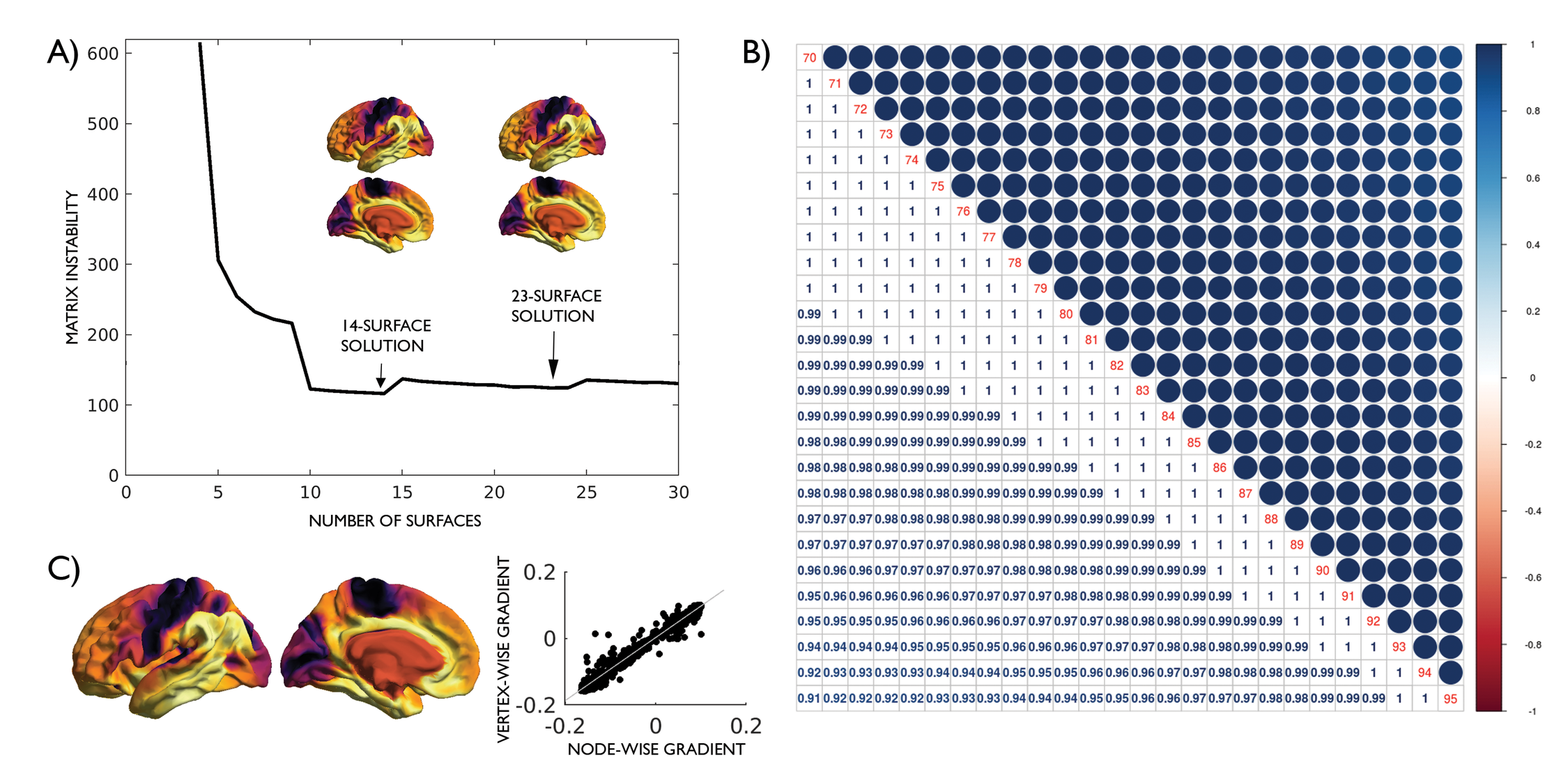

Supplement: S10 Fig — (A) MPCMRI matrix instability using between 4 and 30 intracortical surfaces. G1MRI was consistent regardless of the number of intracortical surfaces used, as shown by the strong spatial correlation of the 14- and 23-surface solutions (r = 0.98, P < 0.001). (B) Correlation matrix depicting the high correspondence of G1MRI solutions with 70%–95% row-wise matrix thresholding (0.91 < r <1, all P < 0.001). (C) Estimation of G1MRI from 20,464 vertices resulted in a consistent G1MRI to the 1,012-parcel construction pipeline (r = 0.98, P < 0.001). In vivo imaging data is openly available as part of the HCP S900 release (https://www.humanconnectome.org/study/hcp-young-adult/document/900-subjects-data-release). G1, first principal gradient; HCP, Human Connectome Project; MPC, microstructure profile covariance; MRI, magnetic resonance imaging. (TIF) [file pbio.3000284.s010.tif]

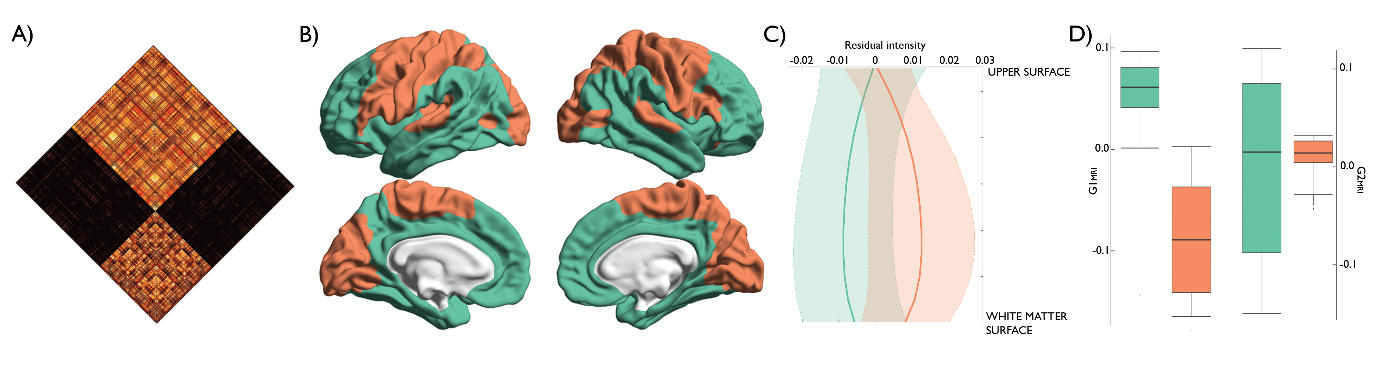

Supplement: S11 Fig — (A) MPCMRI matrix sorted by community membership. (B) Modular decomposition of MPCMRI projected on the conte69 midsurface. (C) Mean ± SD of residual intensity profile for each module, after demeaning. (D) Boxplot depicts the unique positions of modules on the first, but not the second, principal component. In vivo imaging data is openly available as part of the HCP S900 release (https://www.humanconnectome.org/study/hcp-young-adult/document/900-subjects-data-release). HCP, Human Connectome Project; MPC, microstructure profile covariance; MRI, magnetic resonance imaging. (TIF) [file pbio.3000284.s011.tif]

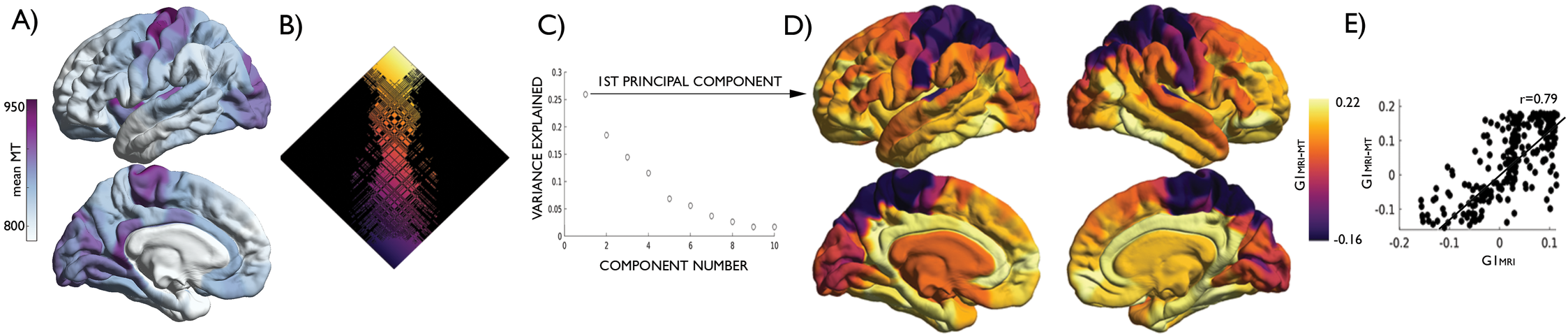

Supplement: S12 Fig — (A) Mean MT intensity across subjects projected onto the cortical surface. (B) Gradient ordered normalised angle matrix. (C) Variance explained by embedding components. (D) G1MRI-MT projected onto conte69 midsurface. (E) Scatterplot depicting the strong correlation between the G1MRI (reconstructed with the 308 parcellation scheme) and G1MRI-MT (r = 0.79, P < 0.001). MT metadata was acquired from the github repository (https://github.com/KirstieJane/NSPN_WhitakerVertes_PNAS2016). G1, first principal gradient; MRI, magnetic resonance imaging; MT, magnetisation transfer. (TIF) [file pbio.3000284.s012.tif]

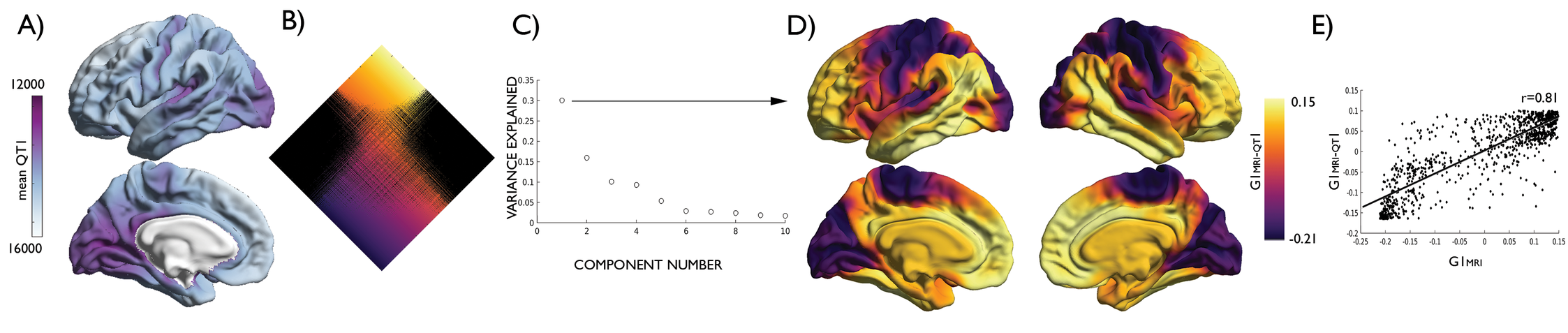

Supplement: S13 Fig — (A) Mean qT1 intensity across subjects projected onto the cortical surface. (B) Gradient ordered normalised angle matrix. (C) Variance explained by embedding components. (D) G1MRI-QT1 projected onto conte69 midsurface. (E) Scatterplot depicting the strong correlation between the G1MRI and G1MRI-QT1 (r = 0.81, P < 0.001). qT1 metadata was acquired in-house and is available on the Github (https://github.com/MICA-MNI/micaopen/tree/master/MPC). G1, first principal gradient; MRI, magnetic resonance imaging; qT1, quantitative T1 relaxometry. (TIF) [file pbio.3000284.s013.tif]
