# Supplementary material for: Microstructural and functional gradients are increasingly dissociated in transmodal cortices
Source: PLoS Biol. 2019 May 20;17(5):e3000284. doi: 10.1371/journal.pbio.3000284 (PMC6544318; doi:10.1371/journal.pbio.3000284)
Supplement: S1 Table — (PDF) [file pbio.3000284.s014.pdf]

Mesulam, 2000	Regional names [1]	Brodmann, 1909 [1]	Von Economo and Koskinas, 1925
Idiotypic primary	Striate	17	OB, OC
	Auditory	41, 42	TC
	Somatosensory	3a, 3b, 1, 2	PA, PB, PC
	Motor	4,6	FA
Modality-specific unimodal	Upstream peristriate	18, 19	OA
	Inferotemporal	20, 21, 37	PH, TE
	Superior temporal	22	TB, TD
	Superior parietal lobule	5, anterior 7	Part of PE
	Inferior parietal lobule	anterior 40	Part of PF
	Premotor	anterior 6, posterior 8, 44	FB, FCBm
Higher-order heteromodal	Prefrontal cortex	9, 10, 45, 46, 47, anterior 11, anterior 12, anterior 8	FC, FD, FDdelta, FDT, FE
	Posterior parietal	posterior 7, 39, 40	PD, PG, parts of PE &PF
	Lateral temporal	parts of 21 & 37	Part TE
	Parahippocampal	parts of 36 & 37	TF
Paralimbic	Orbitofrontal cortex	posterior 11, posterior 12, 13	FF, FG, FH, FJK, FLMH
	Insula	14, 15, 16	IA, IB
	Temporal pole	38	TG
	Parahippocampal	27, 28, 35	HA, HB, HC
	Cingulate	23, 24, 25, 26, 29, 30, 31, 32, 33	LA1, LA2, LC1, LC2, LC3, LD, LE

[1] Ascribed by Mesulam, (2000)

Note: Mesulam (2013) defines transmodal cortex as areas without modality-specific input, thus including heteromodal and paralimbic isocortex.
