# Supplementary material for: Microstructural and functional gradients are increasingly dissociated in transmodal cortices
Source: PLoS Biol. 2019 May 20;17(5):e3000284. doi: 10.1371/journal.pbio.3000284 (PMC6544318; doi:10.1371/journal.pbio.3000284)
Supplement: S2 Table — G1, first principal gradient; HIST, histology-based. (PDF) [file pbio.3000284.s015.pdf]

	Estimate	Std. Error	t value	Pr(>|t|)
Mesulam: idiotypic	0.05651	0.004813	11.74	7.032e-30
Mesulam: unimodal	-0.01105	0.003903	-2.832	0.004723
Mesulam: heteromodal	-0.005509	0.003352	-1.644	0.1006
Mesulam: paralimbic	-0.04945	0.006359	-7.777	1.867e-14
