# Supplementary material for: Microstructural and functional gradients are increasingly dissociated in transmodal cortices
Source: PLoS Biol. 2019 May 20;17(5):e3000284. doi: 10.1371/journal.pbio.3000284 (PMC6544318; doi:10.1371/journal.pbio.3000284)
Supplement: S3 Table — G1, first principal gradient; HIST, histology-based. (PDF) [file pbio.3000284.s016.pdf]

	Estimate	Std. Error	t value	Pr(>|t|)
Von Economo: motor	0.07335	0.008904	8.237	5.589e-16
Von Economo: association[1]	-0.0005984	0.003267	-0.1832	0.8547
Von Economo: association[2]	-0.02703	0.00537	-5.034	5.714e-07
Von Economo: secondary sensory	0.017	0.004712	3.607	0.000325
Von Economo: primary sensory	0.04783	0.008826	5.419	7.53e-08
Von Economo: limbic	-0.06474	0.007497	-8.636	2.32e-17
Von Economo: insular	-0.02524	0.01259	-2.004	0.04531

[1] Frontal and temporal association areas, displayed in yellow (**FIGURE 2**)
[1] Parietal and superior temporal association, displayed in purple (**FIGURE 2**)
