# Supplementary material for: Microstructural and functional gradients are increasingly dissociated in transmodal cortices
Source: PLoS Biol. 2019 May 20;17(5):e3000284. doi: 10.1371/journal.pbio.3000284 (PMC6544318; doi:10.1371/journal.pbio.3000284)
Supplement: S4 Table — G1, first principal gradient; MRI, magnetic resonance imaging. (PDF) [file pbio.3000284.s017.pdf]

	Estimate	Std. Error	t value	Pr(>|t|)
Mesulam: idiotypic	-0.06807	0.005734	-11.87	1.76e-30
Mesulam: unimodal	-0.03628	0.003094	-11.73	7.847e-30
Mesulam: heteromodal	0.01026	0.003598	2.851	0.004444
Mesulam: paralimbic	0.09981	0.004438	22.49	8.103e-91
