# Supplementary material for: Microstructural and functional gradients are increasingly dissociated in transmodal cortices
Source: PLoS Biol. 2019 May 20;17(5):e3000284. doi: 10.1371/journal.pbio.3000284 (PMC6544318; doi:10.1371/journal.pbio.3000284)
Supplement: S5 Table — G1, first principal gradient; MRI, magnetic resonance imaging. (PDF) [file pbio.3000284.s018.pdf]

	Estimate	Std. Error	t value	Pr(>|t|)
Von Economo: motor	0.1486	0.0086	17.28	1.251e-58
Von Economo: association[1]	-0.01441	0.003227	-4.467	8.831e-06
Von Economo: association[2]	0.001541	0.005227	0.2948	0.7682
Von Economo: secondary sensory	-0.008014	0.004666	-1.718	0.08619
Von Economo: primary sensory	0.0974	0.008251	11.8	3.57e-30
Von Economo: limbic	-0.07451	0.007277	-10.24	1.865e-23
Von Economo: insular	-0.05544	0.01284	-4.316	1.746e-05

[1] Frontal and temporal association areas, displayed in yellow (**FIGURE 2**)
[1] Parietal and superior temporal association, displayed in purple (**FIGURE 2**)
