# Supplementary material for: Microstructural and functional gradients are increasingly dissociated in transmodal cortices
Source: PLoS Biol. 2019 May 20;17(5):e3000284. doi: 10.1371/journal.pbio.3000284 (PMC6544318; doi:10.1371/journal.pbio.3000284)
Supplement: S6 Table — FUNC, functional; G1, first principal gradient; MRI, magnetic resonance imaging. (PDF) [file pbio.3000284.s019.pdf]

**Functional community**	**t-statistic**	**2.5% CI**	**97.5% CI**
Visual	-8.3215	-76.998	-47.508
Somatomotor	-10.32	-78.836	-53.551
Dorsal attention	-4.6335	-35.345	-14.249
Salience	-29.099	-202.85	-177.11
Limbic	-22.42	-115.27	-96.641
Frontoparietal	30.123	135.8	154.82
Default mode	54.445	165.76	178.21
